# Publisher Correction: Molecular basis of microhomology-mediated end-joining by purified full-length Polθ

**DOI:** 10.1038/s41467-020-15551-y

**Published:** 2020-04-09

**Authors:** Samuel J. Black, Ahmet Y. Ozdemir, Ekaterina Kashkina, Tatiana Kent, Timur Rusanov, Dejan Ristic, Yeonoh Shin, Antonio Suma, Trung Hoang, Gurushankar Chandramouly, Labiba A. Siddique, Nikita Borisonnik, Katherine Sullivan-Reed, Joseph S. Mallon, Tomasz Skorski, Vincenzo Carnevale, Katsuhiko S. Murakami, Claire Wyman, Richard T. Pomerantz

**Affiliations:** 1 Fels Institute for Cancer Research, Department of Medical Genetics and Molecular Biochemistry, Temple University Lewis Katz School of Medicine, Philadelphia, PA 19140 USA; 2 Department of Molecular Genetics and Department of Radiation Oncology, Erasmus University Medical Center, 3000 CA Rotterdam, The Netherlands; 3 Department of Biochemistry and Molecular Biology, The Center for RNA Molecular Biology, Pennsylvania State University, University Park, PA 16802 USA; 4 Institute for Computational Molecular Science, Temple University, Philadelphia, PA USA; 5 Fels Institute for Cancer Research, Department of Microbiology and Immunology, Temple University Lewis Katz School of Medicine, Philadelphia, PA 19140 USA

**Keywords:** DNA repair enzymes, Double-strand DNA breaks

Correction to: *Nature Communications* 10.1038/s41467-019-12272-9, published online 27 September 2019.

The original version of this Article contained errors in Figure 6. In panel o, the labels incorrectly stated ‘Poleθ’ and “Poleθ + DNA” and should be labelled “Polθ” and “Polθ + DNA”.

In the result section, in the sub-section entitled “Polθ Promotes MMEJ of Long ssDNA”, the sentence “Importantly, the ability of Polθ-pol to perform MMEJ on short (≤12 nt) ssDNA (Fig. 1p, left; Supplementary Fig. 3D and 3E), and short (≤15 nt) overhangs, demonstrates it performs interstrand pairing without Polθ-hel”. should read as follow: “Importantly, the ability of Polθ-pol to perform MMEJ on short (≤12 nt) ssDNA (Fig. 1p, left; Supplementary Fig. 3D and 3E), and short (≤15 nt) overhangs, demonstrates that it performs interstrand pairing without Polθ-hel”.

In the sub-section entitled “Preventing Intrastrand Pairing Stimulates MMEJ by Polθ-Pol”, the sentence “We predicted that preventing base-pairing opportunities between 3′ terminal bases and bases upstream along long the 5′ region of long ssDNA substrates would suppress intrastrand pairing and enable interstrand pairing by Polθ-pol (Fig. 3c)”. should read as follows: “We predicted that preventing base-pairing opportunities between 3′ terminal bases and bases upstream along the 5′ region of long ssDNA substrates would suppress intrastrand pairing and enable interstrand pairing by Polθ-pol (Fig. 3c)”.

In the method section, in the “Proteins” sub-section the sentence “Polθ-pol, Polθ-hel and RPA were purified as described”. should read as follows: “Polθ-pol and Polθ-hel were purified as described”. These corrections have now been included in the HTML and pdf of the article.

Additionally, a technical problem during the publication process resulted in loss of image quality in Figs. 1, 3 and 4. This has now been corrected in both the PDF and HTML versions of the Article.

