# Supplementary material for: Molecular basis of microhomology-mediated end-joining by purified full-length Polθ
Source: Nat Commun. 2019 Sep 27;10:4423. doi: 10.1038/s41467-019-12272-9 (PMC6764996; doi:10.1038/s41467-019-12272-9)
Supplement: Supplementary file 1 — Supplementary Information [file 41467_2019_12272_MOESM1_ESM.pdf]

Molecular Basis of Microhomology-Mediated End-Joining by Purified Full-Length Polθ

Black et al.

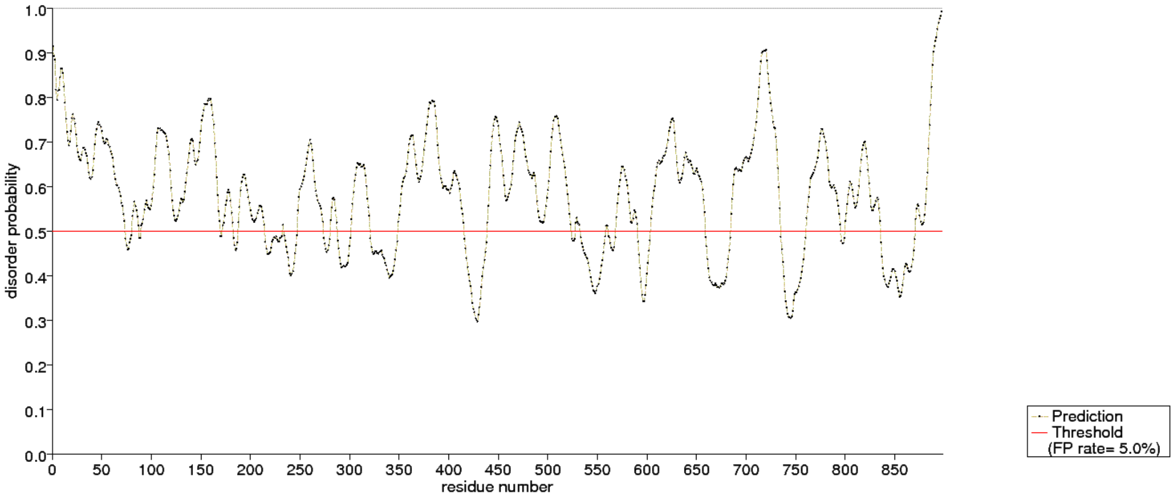

Supplementary Figure 1. Polθ-cen is highly disordered.

Analysis of human Polθ central domain consisting of amino acids 895 to 1791 using Protein Disorder Prediction System (PrDOS). Plot shows disordered probability for each residue. Residue 1 refers to residue 895 in Polθ-cen. Ishida, T and Kinoshita, K, PrDOS: prediction of disordered protein regions from amino acid sequence., *Nucleic Acids Res*, **35**, Web Server issue, 2007.

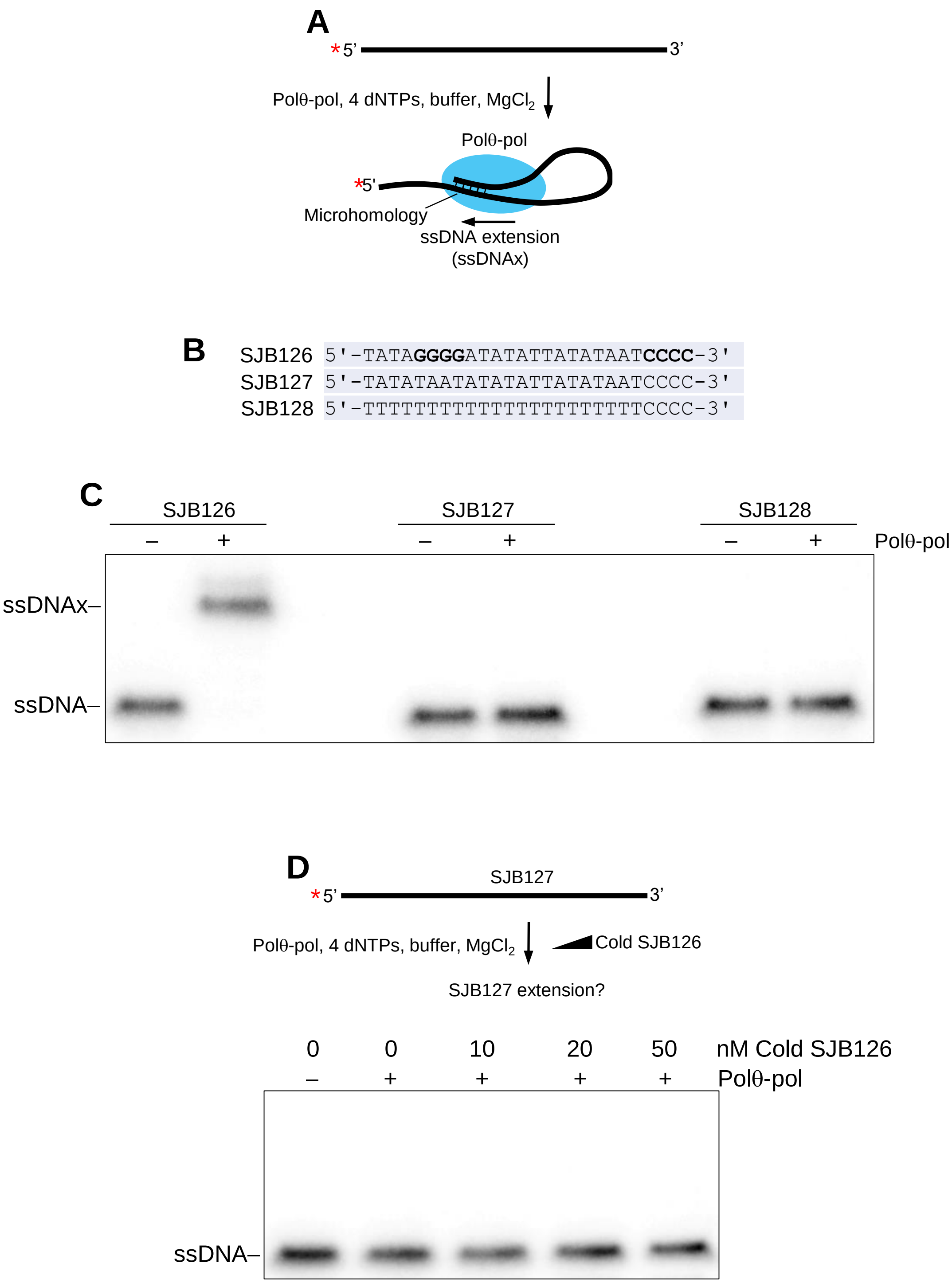

Supplementary Figure 2. Polθ-pol only performs ssDNAx when intrastrand base-pairing is available.
(A) Schematic of Polθ-pol mediated ssDNAx. (B) Sequences of ssDNA substrates used in Polθ-pol mediated ssDNAx assay. (C and D) Denaturing gels showing ssDNAx by Polθ-pol on the indicated ssDNA. Polθ-pol fails to perform ssDNAx due to lack of intrastrand pairing.

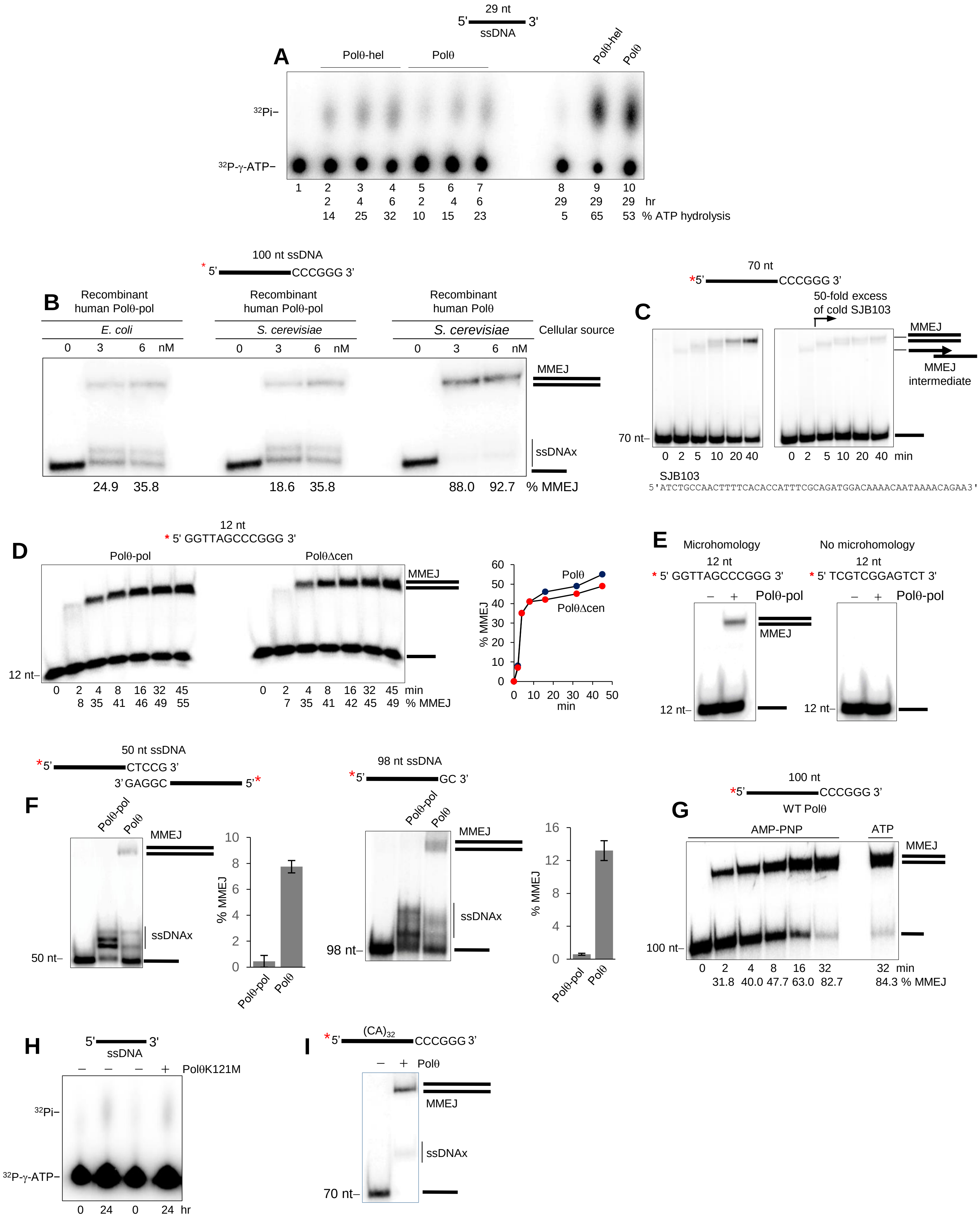

A
29 nt
5' 3'
ssDNA
Polθ-hel
Polθ
Polθ-hel
Polθ
32Pi–
32P-γ-ATP–
1 2 3 4 5 6 7 8 9 10
2 4 6 2 4 6 29 29 29 hr
14 25 32 10 15 23 5 65 53 % ATP hydrolysis
B
100 nt ssDNA
* 5' CCCGGG 3'
Recombinant human Polθ-pol
E. coli
Recombinant human Polθ-pol
S. cerevisiae
Recombinant human Polθ
S. cerevisiae
Cellular source
0 3 6 nM
MMEJ
ssDNAx
24.9 35.8 18.6 35.8 88.0 92.7 % MMEJ
C
70 nt
*5' CCCGGG 3'
50-fold excess of cold SJB103
MMEJ
MMEJ intermediate
70 nt–
0 2 5 10 20 40 0 2 5 10 20 40 min
SJB103
5'ATCTGCCAACTTTTCACACCATTTCGCAGATGGACAAAACAATAAAACAGAA3'
D
12 nt
* 5' GGTTAGCCCGGG 3'
Polθ-pol
PolθΔcen
MMEJ
12 nt–
0 2 4 8 16 32 45 min
8 35 41 46 49 55
0 2 4 8 16 32 45 min
7 35 41 42 45 49 % MMEJ
Polθ
PolθΔcen
% MMEJ
min
E
Microhomology
12 nt
* 5' GGTTAGCCCGGG 3'
No microhomology
12 nt
* 5' TCGTCGGAGTCT 3'
– + Polθ-pol
MMEJ
12 nt–
F
50 nt ssDNA
*5' CTCCG 3'
3' GAGGC 5'*
Polθ-pol
Polθ
MMEJ
ssDNAx
50 nt–
% MMEJ
98 nt ssDNA
*5' GC 3'
98 nt–
G
100 nt
*5' CCCGGG 3'
WT Polθ
AMP-PNP
ATP
MMEJ
100 nt–
0 2 4 8 16 32 32 min
31.8 40.0 47.7 63.0 82.7 84.3 % MMEJ
H
5' 3'
ssDNA
– – – + PolθK121M
32Pi–
32P-γ-ATP–
0 24 0 24 hr
I
*5' (CA)32 CCCGGG 3'
– + Polθ
MMEJ
ssDNAx
70 nt–

Supplementary Figure 3. Control assays for WT Polθ and Polθ variants.

(A) Thin layer chromatography plate image showing a time course of Polθ and Polθ-hel ATPase activities in the presence of ssDNA. Lanes 8-10 demonstrate substantial ^{32}P-γ-ATP hydrolysis over 29 hr. (B) Non-denaturing gel showing MMEJ by Polθ and Polθ-pol purified from both *E. coli* and S. *cerevisiae* on the indicated 100 nt ssDNA. Activity of Polθ-pol is identical regardless of source. (C) Polθ MMEJ processivity assay. A 50-fold excess of the indicated unlabeled ssDNA was added after 2 min (right). Conversion of the intermediate MMEJ products to full-length MMEJ products following addition of the excess cold ssDNA demonstrates processive MMEJ after the initial extension step. (D) Non-denaturing gel showing a time course of MMEJ by the indicated proteins on the indicated 12 nt ssDNA with microhomology (left). Plot of MMEJ time courses (right). (E) Non-denaturing gel of Polθ-pol MMEJ reactions performed on 12 nt ssDNA with (left) and without (right) microhomology. (F) Non-denaturing gels showing MMEJ by the indicated proteins on the indicated ssDNA (left). Bar charts showing % MMEJ. n = 3 +/-s.d. (right). (G) Non-denaturing gel showing MMEJ by Polθ on the indicated 100 nt ssDNA in the presence of AMP-PNP and ATP. Reactions were terminated at the indicated times. (H) PolθK121M lacks ATPase activity. Thin layer chromatography plate image showing ATP hydrolysis after the indicated time intervals in the presence of ssDNA and with and without PolθK121M. (I) Non-denaturing gel showing MMEJ by Polθ on the indicated 70 nt ssDNA which is not predicted to form secondary structures.

A

SJB108

5′ GTTCTTCGGTCTCGAGGTGACTACAAGGATGACGACGACAAGGGCACTGTGAGCTTAGGGTTAGCCCGGG 3′

SJB108 reverse complement

3′ CCCGGGCTAACCCTAAGCTCACAGTGCCCTTGTCGTCGTCATCCTTGTAGTCACCTCGAGACCGAAGAAC 5′

SJB116

5′ CACTGTGAGCTTAGGGTTAGGCGGCTTGCAGAGCACAGAGGCCGCAGAATGTGCTCTAGATTCCGATGCTGACTTGCTGGGTATTATATGTGTGCCCGGG 3′

SJB116 reverse complement

3′ CCCGGGCACACATATAATACCCAGCAAGTCAGCATCGGAATCTAGAGCACATTCTGCGGCCTCTGTGCTCTGCAAGCCGCCTAACCCTAAGCTCACAGTG 5′

B

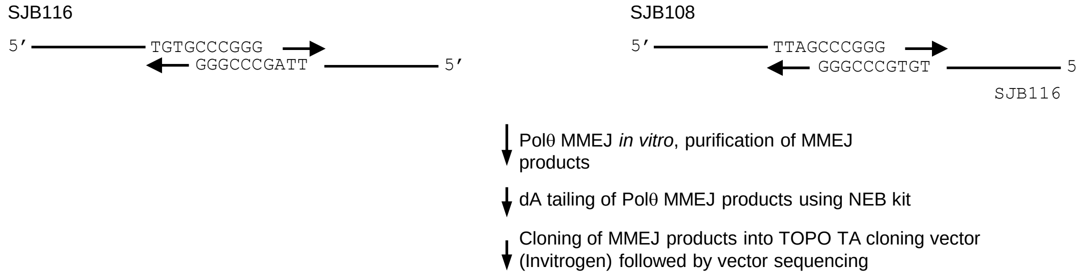

C

Clone 1: SJB116 - SJB108 reverse complement sequencing

NNNNNNNNNNTAGGGCGATTGGGCCCTCTAGATGCATGCTCGAGCGGCCGCCAGT
GTGATGGATATCTGCAGAATTCGCCCTT**CACTGTGAGCTTAGGGTTAGGCGGCTT**
GCAGAGCACAGAGGCCGCAGAATGTGCTCTAGATTCCGATGCTGACTTGCTGGGT
ATTATATGTGTGCCCGGGCTAACCCTAAGCTCACAGTGCCCTTGTCGTCGTCATC
CTTGTAGTCACCTCGAGACCGAAA**AAC**AAGGGCGAATTCCAGCACACTGGCGGCC
GTTACTAGTGGATCCGAGCTCGGTACCAAGCTTGGCGTAATCATGGTCATAGCTG

Clone 18: SJB116 - SJB108 reverse complement sequencing

NNNNNNNNNNNNNNGGGCGATTGGGCCCTCTAGATGCATGCTCGAGCGGCCGCCAG
TGTGATGGATATCTGCAGAATTCGCCCTT**CACTGTGAGCTTAGGGTTA**A**GCGGCT**
TGCAGAGCACAGAGGCCGCAGAATGTGCTCTAGATTCCGATGCTGACTTGCTGGG
TATTATATGTGTGCCCGGGCTAACCCTAAGCTCACAGTGCCCTTGTCGTCGTCAT
CCTTGTAGTCACCTCGAGACCGAAGAACAAGGGCGAATTCCAGCACACTGGCGGC
CGTTACTAGTGGATCCGAGCTCGGTACCAAGCTTGGCGTAATCATGGTCATAGCT

Clone 2: SJB108 - SJB116 reverse complement sequencing

NNNNNNNNNNNNAGGGCGATTGGGCCCTCTAGATGCATGCTCGAGCGGCCGCCAGT
GTGATGGATATCTGCAGAATTCGCCCTT**GTTCTTCGGTCTCGAGGTGACTACAAG**
GATGACGACGACAAGGGCACTGTGAGCTTAGGGTTAGCCCGGGCACACATATAAT
ACCCAGCAAGTCAGCATCGGAATCTAGAGCACATTCTGCGGCCTCTGTGCTCTGC
AAGCCGCCTAACCCTAAGCTCACAGTGAAGGGCGAATTCCAGCACACTGGCGGCC
GTTACTAGTGGATCCGAGCTCGGTACCAAGCTTGGCGTAATCATGGTCATAGCTG

Clone 4: SJB108 - SJB116 reverse complement sequencing

NNNNNNNNNNNNNNGGGCGATTGGGCCCTCTAGATGCATGCTCGAGCGGCCGCCAG
TGTGATGGATATCTGCAGAATTCGCCCTT**GTTCTTCGGTCTCGAGGTGACTACAA**
GGATGACGACGACAAGGGCACTGTGAGCTTAGGGTTAGCCCGGGCACACATATAA
TACCCAGCAAGTCAGCATCGGAATCTAGAGCACATTCTGCGGCCTCTGTGCTCTG
CAAGCCGCCTAACCCTAAGCTCACAGTGAAGGGCGAATTCCAGCACACTGGCGGC
CGTTACTAGTGGATCCGAGCTCGGTACCAAGCTTGGCGTAATCATGGTCATAGCT

D

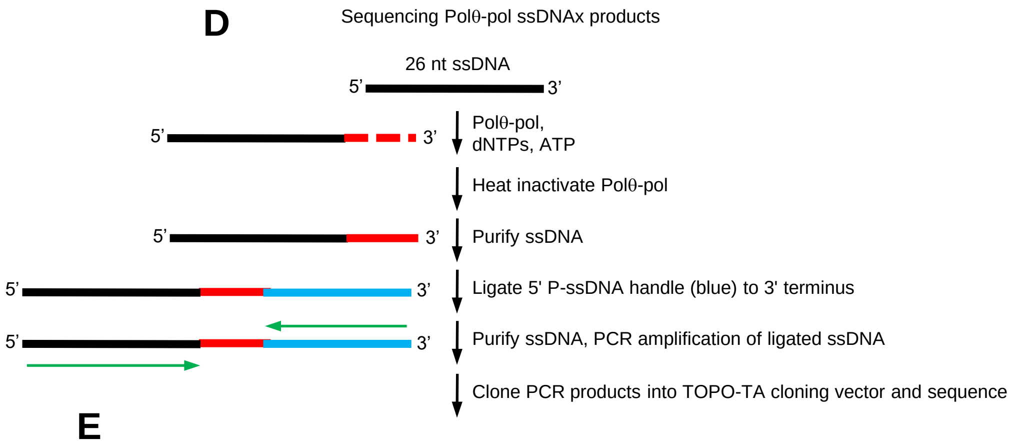

E

ssDNA sequence (5'-3')	ssDNAx
CACTGTGAGCTTAGGGTTAGCCCGGG	CTAGCAGTGA
CACTGTGAGCTTAGGGTTAGCCCGGG	TCA
CACTGTGAGCTTAGGGTTAGCCCGGG	TGA
CACTGTGAGCTTAGGGTTAGCCCGGG	T
CACTGTGAGCTTAGGGTTAGCCCGGG	T
CACTGTGAGCTTAGGGTTAGCCCGGG	T
CACTGTGAGCTTAGGGTTAGCCCGGG	T

F

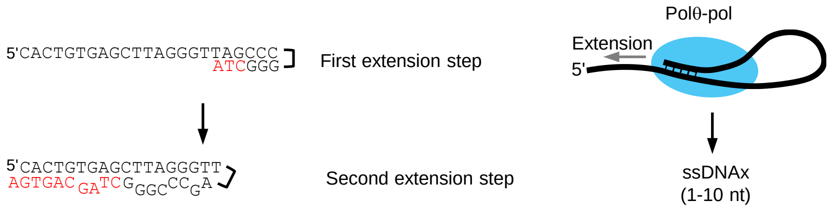

Supplementary Figure 4. Sequencing of Polθ MMEJ products and Polθ-pol ssDNA extension products.

(A) Sequences (and reverse complements) of ssDNA substrates (SJB108, SJB116) used in Polθ MMEJ assay. (B) Schematic of expected MMEJ via the 6 bp (5'-CCCGGG-3') microhomology tract (top left and right). Schematic of procedure used for sequencing Polθ MMEJ products (bottom). (C) Representative plasmid clones containing expected Polθ MMEJ products. Bold black, red and blue text corresponds to the colored sequences in panel a. Yellow highlight indicates mutation. (D) Schematic of method used for sequencing Polθ-pol ssDNAx products. (E) Representative sequences of Polθ-pol ssDNAx products. Initial ssDNA substrate sequence (black). Nucleotides transferred to the 3' terminus of ssDNA by Polθ-pol (red; ssDNAx). (F) Model of Polθ-pol ssDNAx via intrastrand pairing and extension. The model predicts how the first sequence in panel e is generated by Polθ-pol. Because Polθ-pol is highly promiscuous various intrastrand non-canonical base-pairing options may lead to the observed ssDNAx events in panel e. Figure S2 unequivocally shows that Polθ-pol ssDNAx is dependent on intrastrand pairing *in cis*.

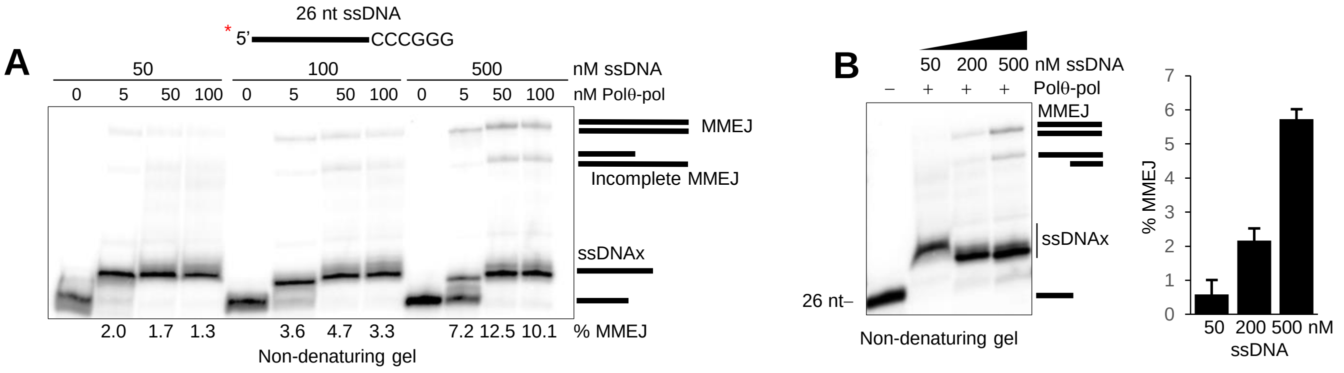

Supplementary Figure 5. Increasing concentrations of ssDNA slightly stimulates MMEJ by Polθ-pol.
(A) Non-denaturing gel showing MMEJ by Polθ-pol on ssDNA with increasing concentrations of substrate and enzyme. (B) Non-denaturing gel showing MMEJ by Polθ-pol (left). Bar chart showing % MMEJ. n = 3 +/-s.d. (right).

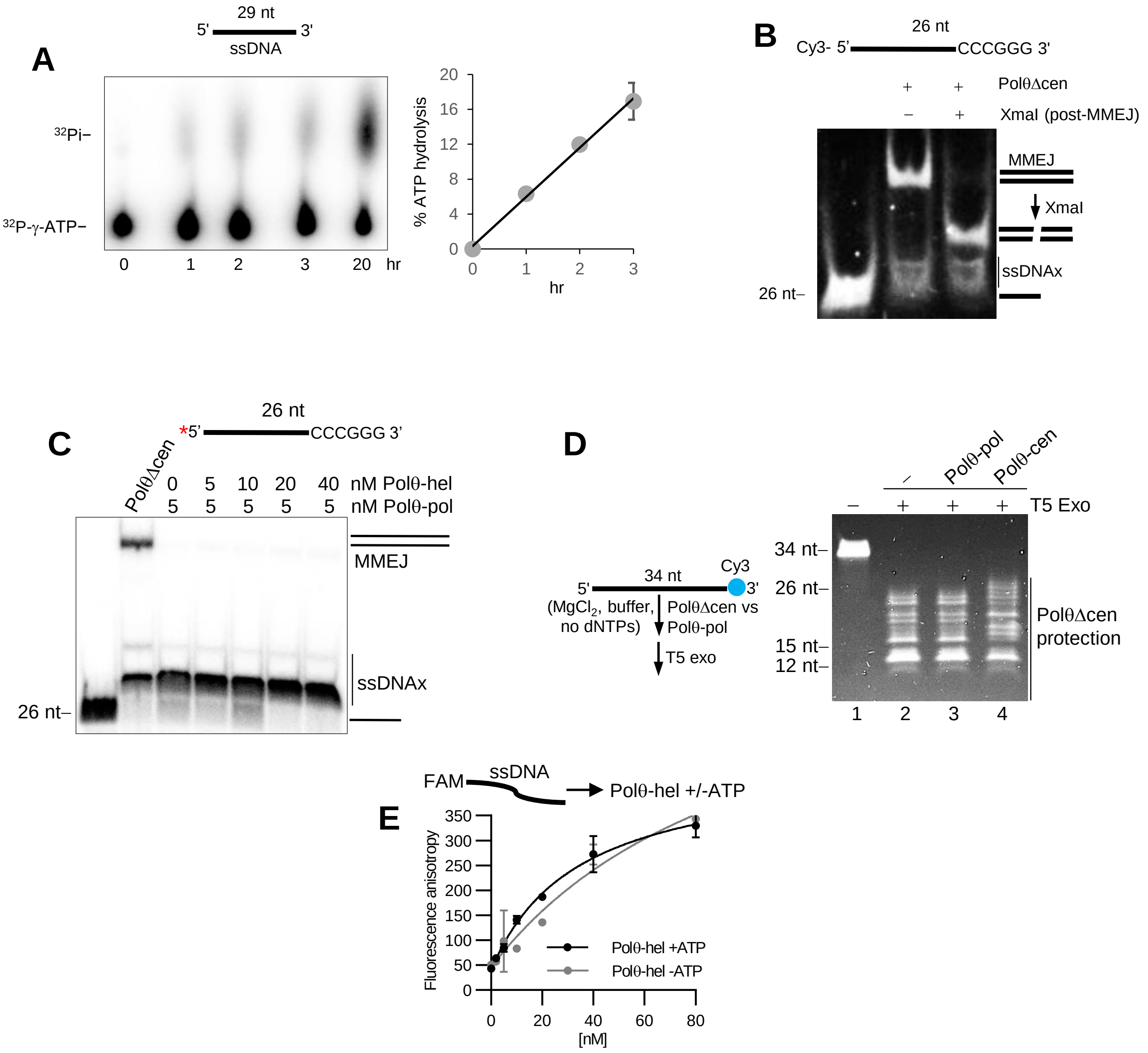

Supplementary Figure 6. Control assays for PolθΔcen and Polθ-hel.

(A) Thin layer chromatography plate image showing a time course of PolθΔcen ATPase activity in the presence of ssDNA (left). Plot of PolθΔcen ATPase activity. n = 3 +/-s.d. (right). (B) Control showing MMEJ of 26 nt ssDNA by PolθΔcen. Non-denaturing gel showing PolθΔcen MMEJ of the indicated ssDNA (lane 2), followed by digestion of the MMEJ product by XmaI (lane 3) which cleaves CCCGGG double-strand DNA. (C) Non-denaturing gel showing MMEJ reactions performed with the indicated proteins on 26 nt ssDNA with the indicated 6 bp microhomology. (D) Exonuclease footprinting of PolθΔcen on ssDNA. Schematic of assay (left). Denaturing gel showing T5 exonuclease (0.5 U/ul) digestion of the indicated ssDNA with 10 nM Polθ-pol (lane 3), 10 nM PolθΔcen (lane 4), or no protein (lane 2). (E) ATP has no significant effect on Polθ-hel ssDNA binding. Plot showing fluorescence anisotropy in the presence of the indicated amounts of Polθ-hel with and without ATP. n = 3 +/-s.d.

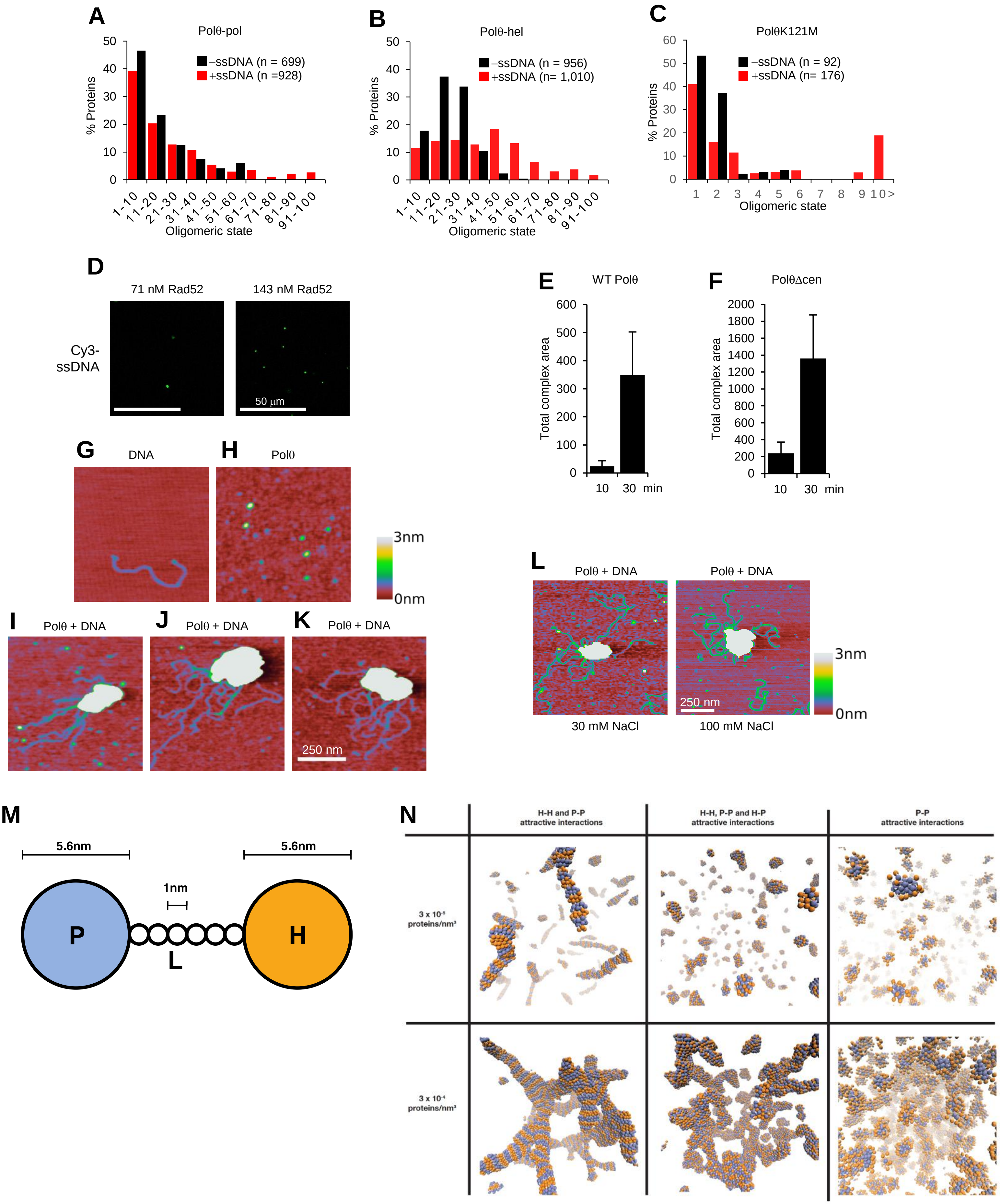

A
Polθ-pol
% Proteins
–ssDNA (n = 699)
+ssDNA (n =928)
Oligomeric state
B
Polθ-hel
–ssDNA (n = 956)
+ssDNA (n= 1,010)
C
PolθK121M
–ssDNA (n = 92)
+ssDNA (n= 176)
D
71 nM Rad52
143 nM Rad52
Cy3-ssDNA
50 µm
E
WT Polθ
Total complex area
10 30 min
F
PolθΔcen
G
DNA
H
Polθ
3nm
0nm
I
Polθ + DNA
J
Polθ + DNA
K
Polθ + DNA
250 nm
L
Polθ + DNA
Polθ + DNA
30 mM NaCl
100 mM NaCl
M
5.6nm
5.6nm
1nm
P
L
H
N
H-H and P-P attractive interactions
H-H, P-P and H-P attractive interactions
P-P attractive interactions
3 x 10⁻⁵ proteins/nm³
3 x 10⁻⁴ proteins/nm³

Supplementary Figure 7. Supplementary SFM, confocal imaging, and computational modeling.

(A-C) Bar charts showing oligomeric states of indicated proteins with (red) and without (black) 26 nt ssDNA determined by SFM volume measurements. (D) Confocal microscopy images of 46 nt Cy3-ssDNA with Rad52 at 71 nM and 143 nM (as heptamer). Scale bar = 50 μm. (E and F) Bar charts showing total WT Polθ (E) and PolθΔcen (F) Cy3-ssDNA complex area formed after indicated times. n = 8 +/-s.d. (G-K) SFM images of DNA (G), Polθ (H), and Polθ-DNA complexes (I-K). DNA length, 1.8 kb. Scale bar = 250 nm. (L) SFM images of Polθ-DNA complexes in 30 mM and 100 mM NaCl. DNA length, 1.8 kb. Scale bar = 250 nm. (M) Physical model for PolθΔcen is modelled by two large spheres, representing the polymerase [P] and helicase [H] domains, connected by a short flexible polymer chain made by 6 beads, representing the $(GGGGS)^3$ linker [L]. (N) Helicase or polymerase attractive interactions. Assembly differences at two protein densities using selectively attractive interactions between H-H, P-P and H-P. H-H and P-P attractive interactions yield elongated characteristic shapes, which are less abundant adding H-P attraction. Having only P-P attraction yield no gel formation at the chosen densities.
