# Supplementary material for: Molecular basis of microhomology-mediated end-joining by purified full-length Polθ
Source: Nat Commun. 2019 Sep 27;10:4423. doi: 10.1038/s41467-019-12272-9 (PMC6764996; doi:10.1038/s41467-019-12272-9)
Supplement: Supplementary file 3 — Description of Additional Supplementary Files [file 41467_2019_12272_MOESM3_ESM.pdf]

**Description of Additional Supplementary Files**

File Name: Supplementary Movie 1
Description: Visualization of Polθ promoting Cy3-DNA accumulation
100 nM RP334Cy3, 3 nM Polθ, 10 min at room temperature, 2 min capture at room temperature.

File Name: Supplementary Movie 2
Description: Visualization of PolθΔcen promoting Cy3-DNA accumulation
100 nM RP334Cy3, 15 nM PolθΔcen, 10 min capture at room temperature, captured after the addition of PolθΔcen continuously.

File Name: Supplementary Movie 3
Description: Dynamic particles of MMEJ in real time by PolθΔcen
40 nM RP344Cy3, 15 nM PolθΔcen, highlighting the dynamic nature of the particles captured after the addition of PolθΔcen.

File Name: Supplementary Movie 4
Description: MMEJ in real time by PolθΔcen
40 nM RP344Cy3, 15 nM PolθΔcen, 44 min capture at 37C, captured after the addition of PolθΔcen continuously.

File Name: Supplementary Movie 5
Description: Simulation of protein aggregation
Movie depicting the aggregation of PolθΔcen in a box of size 125nm and at a volume fraction of 0.05, starting from a random initial configuration.

File Name: Supplementary Movie 6
Description: Protein cluster structure
Final configuration of Supplementary Movie 5 visualized from different angles.

File Name: Supplementary Movie 7
Description: Simulation of protein aggregation
Movie depicting the aggregation of PolθΔcen in a box of size 250nm and at a volume fraction of 0.05, starting from a random initial configuration.

File Name: Supplementary Movie 8
Description: Protein cluster structure
Final configuration of Supplementary Movie 7 visualized from different angles.
