# Supplementary material for: Molecular basis of microhomology-mediated end-joining by purified full-length Polθ
Source: Nat Commun. 2019 Sep 27;10:4423. doi: 10.1038/s41467-019-12272-9 (PMC6764996; doi:10.1038/s41467-019-12272-9)
Supplement: Supplementary file 12 — Reporting Summary [file 41467_2019_12272_MOESM12_ESM.pdf]

nature research

Corresponding author(s): Richard Pomerantz

Last updated by author(s): Aug 20, 2019

Reporting Summary

Nature Research wishes to improve the reproducibility of the work that we publish. This form provides structure for consistency and transparency in reporting. For further information on Nature Research policies, see Authors & Referees and the Editorial Policy Checklist.

Statistics

For all statistical analyses, confirm that the following items are present in the figure legend, table legend, main text, or Methods section.

n/a	Confirmed	
☐	☒	The exact sample size (*n*) for each experimental group/condition, given as a discrete number and unit of measurement
☐	☒	A statement on whether measurements were taken from distinct samples or whether the same sample was measured repeatedly
☐	☒	The statistical test(s) used AND whether they are one- or two-sided<br>*Only common tests should be described solely by name; describe more complex techniques in the Methods section.*
☒	☐	A description of all covariates tested
☒	☐	A description of any assumptions or corrections, such as tests of normality and adjustment for multiple comparisons
☐	☒	A full description of the statistical parameters including central tendency (e.g. means) or other basic estimates (e.g. regression coefficient) AND variation (e.g. standard deviation) or associated estimates of uncertainty (e.g. confidence intervals)
☐	☒	For null hypothesis testing, the test statistic (e.g. F, t, r) with confidence intervals, effect sizes, degrees of freedom and P value noted<br>*Give P values as exact values whenever suitable.*
☒	☐	For Bayesian analysis, information on the choice of priors and Markov chain Monte Carlo settings
☒	☐	For hierarchical and complex designs, identification of the appropriate level for tests and full reporting of outcomes
☒	☐	Estimates of effect sizes (e.g. Cohen's *d*, Pearson's *r*), indicating how they were calculated

Our web collection on statistics for biologists contains articles on many of the points above.

Software and code

Policy information about availability of computer code

Data collection	DNA resolved in polyacrylamide gels was scanned by phosphorimager (Fujifilm FLA 7000) or autoradiography. Fluorescent DNA gels were scanned using FluorChem Q imager (Alpha Innotech). Confocal microscopy images were obtained by a Leica DMi8 scanning confocal microscope with a 63x objective at the Cy3 emission wavelength. A Clariostar (BMG Labtech) plate reader was used to measure fluorescence anisotropy and FRET. Scanning force microscopy images were obtained on a NanoScope IV SFM (Digital Instruments; Santa Barbara, CA) operating in tapping mode in air with a type J scanner. Electron microscopy images were imaged in a FEI Tecnai BioTwin Spirit transmission electron microscopy at Penn State University Cryo-Electron Microscopy facility. Computational modeling simulations were generated with LAMMPS Molecular Dynamics Simulator software, as described.
Data analysis	The size of proteins was measured from NanoScope scanning force microscopy images imported into IMAGE SXM 1.89 (National Institutes of Health IMAGE version modified by Steve Barrett, Surface Science Research Centre, Univ. of Liverpool, Liverpool, U.K.). Statistical analysis for scanning force microscopy was done using QtiPlot (Version 0.9.8.9 svn 2288) and LibreOffice (Version: 5.1.6.2). Gels containing DNA and protein were analyzed by ImageJ Gel Analysis. Confocal microscopy images were quantified by quantified using ImageJ particle analysis.

For manuscripts utilizing custom algorithms or software that are central to the research but not yet described in published literature, software must be made available to editors/reviewers. We strongly encourage code deposition in a community repository (e.g. GitHub). See the Nature Research guidelines for submitting code & software for further information.

Data

Policy information about availability of data

All manuscripts must include a data availability statement. This statement should provide the following information, where applicable:

- Accession codes, unique identifiers, or web links for publicly available datasets
- A list of figures that have associated raw data
- A description of any restrictions on data availability

The generated data that support these findings are available in the "Source Data" file provided and in the Supplementary Information. Any additional data is available on reasonable request from RTP.

Field-specific reporting

Please select the one below that is the best fit for your research. If you are not sure, read the appropriate sections before making your selection.

☒ Life sciences ☐ Behavioural & social sciences ☐ Ecological, evolutionary & environmental sciences

For a reference copy of the document with all sections, see nature.com/documents/nr-reporting-summary-flat.pdf

Life sciences study design

All studies must disclose on these points even when the disclosure is negative.

Sample size	Sample size was chosen as described in the figure legends to achieve statistical significance.
Data exclusions	No data was excluded.
Replication	Experiments were repeated at least in triplicate, as described in the figure legends, to achieve statistical significance.
Randomization	N/A
Blinding	N/A

Reporting for specific materials, systems and methods

We require information from authors about some types of materials, experimental systems and methods used in many studies. Here, indicate whether each material, system or method listed is relevant to your study. If you are not sure if a list item applies to your research, read the appropriate section before selecting a response.

Materials & experimental systems

n/a	Involved in the study	
☐	☒	Antibodies
☒	☐	Eukaryotic cell lines
☒	☐	Palaeontology
☒	☐	Animals and other organisms
☒	☐	Human research participants
☒	☐	Clinical data

Methods

n/a	Involved in the study	
☒	☐	ChIP-seq
☒	☐	Flow cytometry
☒	☐	MRI-based neuroimaging

Antibodies

Antibodies used	Rabbit anti-Pol theta (ThermoFisher #PA5-69577), Goat Anti-Rabbit IgG (Upstate #12-348)
Validation	Antibodies were tested against purified full-length PolQ protein, PolQ mutants, truncated versions of the protein in addition to different strains of the protein.
